# Fine particulate matter and ischemic heart diseases inrelation to sex. An ecological time series study

**DOI:** 10.1590/1516-3180.2018.0239040119

**Published:** 2019-05-08

**Authors:** Paola Cristina Ribeiro, Luiz Fernando Costa Nascimento, Ana Aparecida Almeida, Marcelo dos Santos Targa, Ana Cristina Gobbo Cesar

**Affiliations:** I BSc. Master’s Student, Postgraduate Program on Environmental Sciences, Universidade de Taubaté (UNITAU), Taubaté (SP), Brazil.; II MD, PhD. Researcher, Postgraduate Program on Environmental Sciences, Universidade de Taubaté (UNITAU), Taubaté (SP), Brazil.; III PhD. Researcher, Postgraduate Program on Environmental Sciences, Universidade de Taubaté (UNITAU), Taubaté (SP), Brazil.; IV PhD. Researcher, Postgraduate Program on Environmental Sciences, Universidade de Taubaté (UNITAU), Taubaté (SP), Brazil; V PhD. Assistant Professor, Instituto Federal de Educação Ciência e Tecnologia de São Paulo (IFSP), Campus Bragança Paulista (SP), Brazil.

**Keywords:** Cardiovascular diseases, Air pollutants, Particulate matter

## Abstract

**BACKGROUND:**

Exposure to some air pollutants is associated with cardiovascular diseases. The objective of this study was to quantify the effect of exposure to fine particulate matter in hospitalizations due to ischemic heart disease and the costs to the healthcare system.

**DESIGN AND SETTING:**

Time-series ecological study conducted in Taubaté, Brazil.

**METHODS:**

Data on hospitalizations due to ischemic heart diseases (ICD I-20 to I-24) in the municipality of Taubaté (SP), Brazil, among adults of both sexes aged 40 years and over, from August 2011 to July 2012, were obtained from DATASUS. Fine particulate matter (PM_2.5_) concentrations were estimated from a mathematical model. Poisson regression was used in statistical analyses to estimate the relative risks of exposure to PM_2.5_ for both sexes and after stratification according to sex. The excess of hospitalizations and consequent excess expenditure for the healthcare system were calculated.

**RESULTS:**

There were 1040 admissions, among which 382 had ischemic heart diseases (257 males). Themean PM_2.5_ concentration was 13.2 µg/m^3^ (SD = 5.6). Significant effects from exposure were noted 4and 5 days after exposure (lag 4 and lag 5) for both sexes and for male sex; for female sex, the effect was 2 days after exposure (lag 2). There were 59 excess hospitalizations for an increase in PM_2.5_ concentration of 5 µg/m^3^ and excess expenditure of US$ 150,000 for the National Health System.

**CONCLUSIONS:**

An excess of hospital admissions due to ischemic heart disease, with excess expenditure, was identified consequent to PM_2.5_ exposure.

## INTRODUCTION

Over the period from August 2011 to July 2012, cardiovascular diseases accounted for 1.1million hospitalizations in Brazil, generating a cost of approximately US$ 1 billion. Inthe state of São Paulo, the most populous and developed state in Brazil, there were approximately 260,000 hospitalizations, costing approximately US$ 300 million. Specifically, the 65,000 hospitalizations due to ischemic heart diseases consumed resources of the order of US$130million.^1^

Cardiovascular diseases are typically associated with factors such as smoking, hypercholesterolemia, systemic arterial hypertension, family history of ischemic diseases of the heart, smoking, obesity and sedentary lifestyle. Moreover, some studies have identified an association between exposure to air pollutants and these diseases. Several positive associations have been found in relation to exposure to fine particulate matter (PM_2.5_), i.e. particles with an aerodynamic diameter of less than 2.5 µ. In urban areas, PM_2.5_ constitutes about 50 to 60% of PM_10_ (particulate material with an aerodynamic diameter of less than 10 µ).^2^ These particles result from burning fuels such as coal, gasoline, oil and biomass; from processes involving high temperatures such as smelting and steel production; and from soil dust. The particles can reach the terminal portionsof the respiratory tree. The material adsorbed onto the particle’s surface depends on the region ofthecity from which it originated.^3^^,^^4^

In the region of the Paraíba valley, in the state of São Paulo, these particles present high ion concentrations of SO_4_^2-^, NH_4_^+^ and K^+^, with long half-lives of the order of days to weeks. Theparticles have the capacity for dispersal over long distances, of the order of 100 to 1000 km.^3^^,^^4^

Higher risk of death due to ischemic heart disease, arrhythmia, heart failure and cardiac arrest has also been correlated with long-term exposure to PM_2.5_.^5^^,^^6^ On the other hand, over the last decade, epidemiological studies have highlighted that the effects of pollution and the risk of cardiovascular diseases are more evident among women than among men,^7^^,^^8^^,^^9^ but without reaching any consensus in this regard.

The PM_2.5_ concentration is usually quantified by state environmental agencies, but this monitoring does not exist in all municipalities or in all states in Brazil. One alternative for estimating these concentrations is to use modeling methods such as the Coupled Chemistry Aerosol and Tracer Transport model for the Brazilian Atmospheric Modeling System (CCATT-BRAMS). This considers the emission and transportation of various aerosol gases and forms of particulate matter, with daily estimates of various pollutants.^10^ The Center for Weather Forecasting and Climate Studies of the Brazilian National Institute for Space Research (Centro de Previsão de Tempo e Estudos Climáticos, Instituto Nacional de Pesquisas Espaciais, CPTEC-INPE) runs this model in an operational manner, producing daily data every three hours, with a horizontal resolution of 25 km by 25 km, 40 meters above ground level, covering all of South America.^10^ This model has been used in some Brazilian studies.^11^^,^^12^

## OBJECTIVE

The objective of this study was to estimate the association between exposure to fine particulate matter (PM_2.5_) and hospitalizations due to ischemic heart disease in the city of Taubaté, a medium-sized city in the state of São Paulo, according to the sex of the patients, using data estimated through mathematical modeling.

## METHODS

A time-series ecological study was carried out in the city of Taubaté, based on estimates of PM_2.5_ concentrations that were obtained from the CCATT-BRAMS daily monitoring system (http://meioambiente.cptec.inpe.br/).

Taubaté is located in the mesoregion of the Paraíba valley, in the state of São Paulo, between the two largest economic axes of Brazil: 130 km from São Paulo and 280 km from Rio de Janeiro. Itsgeographical coordinates are 23°01’S and 45°33’W, and it has an approximate population of 300,000 inhabitants in a territorial area of 625 km^2^. It has a humid subtropical climate and lies alongside the Dutra Highway, which links São Paulo to Rio de Janeiro and is characterized by intense vehicular traffic. It has two hospitals that attend patients within the Brazilian National Health System (Sistema Único de Saúde, SUS).

The data from hospitalizations due to ischemic heart disease that were used in this study related to conditions classified under codes I-20 to I-24 of the International Classification of Diseases, 10^th^ revision (ICD-10). These data covered the period from August 1, 2011, to July 31, 2012, and were collected from the SUS website (DATASUS) (http://www2.datasus.gov.br/DATASUS/index.php?area=0203&id=6926&VObj=http://tabnet.datasus.gov.br/cgi/deftohtm.exe?sih/cnv/ni). The values were organized in columns, separated according to municipality code, date of hospitalization, diagnosis and age. Only adults aged 40 years and over were considered in this study, and these subjects were subsequently stratified as male and female.

The following variables were considered in analyzing the data. The dependent variable was the daily number of hospitalizations due to cardiovascular diseases, obtained from DATASUS. The independent variables were the concentration of the pollutant PM_2.5_ (µg/m^3^), temperature (°C) and relative humidity (%), which were obtained from CPTEC-INPE. Days of the week and long-term seasonality were the control variables.

In studies on the impact of air pollution on health, it is necessary to take into account the short-term trend represented by the days of the week because, at weekends, the number of hospital visits is lower than on weekdays. Long-term seasonality is another important time trend, since meteorological factors and pollutant concentrations vary during the year. Regarding air temperature and relative humidity, these climatic variables are correlated with hospital admission rates and their inclusion changes the coefficients and, consequently, the relative risks in an important way. Itis also important to note that practically all studies on this topic have included these variables, which has made these studies comparable with each other.^5^^,^^6^^,^^7^^,^^8^^,^^9^

The frequency distributions of the different independent variables, i.e. PM_2.5_ concentration, temperature and relative air humidity, and the daily numbers of cases of hospitalization were expressed as means, standard deviations and minimum and maximum values. This was done using the Statistica v.7 software.

The data were analyzed using the Poisson regression generalized additive model because the hospitalizations were numerical data that followed Poisson distribution. This regression is expressed by equation (1):

$$Ln\;(HA)\;=\;\beta_{0}\;+\;\beta_{1}\;(CONC)\;+\;\beta_{2}\;(RH)\;+\;\;\beta_{3}\;(T)\;+\;\beta_{4}\;(SEASON)\;+\;\beta_{5}\;(D)$$ (1)

where:

β’s are regression coefficients;

HA is daily hospital admission;

CONC is the air pollutant concentration;

RH is the relative humidity value;

T is the temperature value;

SEASON is the long-term trend (seasonality); and

D is the day of the week.

This model provided a coefficient (coeff) that could be transformed into the relative risk (RR) of occurrence of the outcome, according to the expression RR = exp^(coeff)^. These RRs were calculated with their respective 95% confidence intervals for hospitalizations due to ischemic heart diseases for females, males and both sexes.

We also tested whether the effect estimates were statistically different between males and females by computing the 95% confidence interval using the following equation (2):

$$Q_{1}\;-\;Q_{2}\;\pm \;1.96\;\sqrt{\left({SE}_{1}^{2}\right)+({SE}_{2}^{2})}$$ (2)

where:

Q_1_ and Q_2_ are coefficients of these categories; and

SE_1_ and SE_2_ are the respective standard errors.^13^

Since the effects of exposure may occur either on the same day or on subsequent days, lags of 0 to 7 days after exposure were taken into consideration.

In these analyses, an increase in exposure to the PM_2.5_ pollutant of 5 µg/m^3^ was calculated to determine the percentage increase (PI) in RR, and this was expressed through equation (3):

PI =[exp (ß * ΔC) -1] * 100 (3)

where:

ß is the value provided by the Poisson regression; and ΔC is the variation in the fine particulate concentration, which in this case was 5 µg/m^3^. Considering that PM_2.5_ constitutes approximately 50% of PM_10_, this value can even be used for comparison purposes with otherstudies.

From this increase in PM_2.5_ concentration, for both sexes and separately for the male and female sexes, we estimated the proportional attributable risk (PAR), which was given by PAR = ­[1-1/­RR]. The excess of hospitalizations was also estimated through the expression PAF = (PAR * N), where PAF is the population attributable fraction and N is the number of hospitalizations for both sexes and separately for the male and female sexes. From this excess number of hospitalizations, it was possible to estimate the excess expenditure in accordance with the number of hospitalizations due to ischemic heart diseases, which was obtained from the DATASUS website.

### Ethical criteria

This study was not evaluated by an internal review board (ethics committee), because the records used are available from DATASUS, which is a public website.

## RESULTS

During the study period, 1040 hospitalizations relating to diseases of the cardiovascular system (ICD-10 codes I-00 to I-99) occurred, among which 903 (87.0%) were cases of individuals aged 40 years and over. There were 382 hospitalizations due to ischemic heart disease among individuals aged 40 years and over in the city of Taubaté (SP) and 257 (67.3%) of these cases were among men.

The pollutant values observed over this period and the hospitalizations due to ischemic heart diseases were expressed as averages, as shown in Table 1. The PM_2.5_ concentrations showed a significant increase from October to July. The safe limit established by the World Health Organization (25 ug/m^3^) was exceeded on eight days, reaching a maximum value of 41 µg/m^3^.

**Table 1.** Daily mean, maximum (Max) and minimum (Min) values andthe respective standard deviations (SD) for PM_2.5_ concentrations (µg/m^3^), temperature (°C), relative humidity (%) and hospitalizations due to ischemic heart diseases. Taubaté, São Paulo, 2011-2012

Variables	Mean	SD	Min	Max
PM_2.5_	13.2	5.6	0.4	41.2
Temperature	22.9	3.9	9.8	38.4
Relative humidity	95.3	8.7	45.0	100.0
Hospitalizations	0.9	0.9	0	4

PM_2.5_ = fine particulate matter.

Table 2 shows the Pearson correlation matrix with the study variables. Table 3 presents the values of the coefficients provided through Poisson regression with the respective standard deviations for hospitalizations due to ischemic heart diseases, with delays of 0 to 7 days after PM_2.5_ exposure, among individuals of both sexes and stratified according to male and female sex. It was seen that admission was significantly associated with exposure to PM_2.5_ in the following situations: unstratified analyses at lag 4 (RR=1.04; 95% CI: 1.01-1.06) and at lag 5 (RR = 1.04; 95% CI: 1.01-1.06); among males at lag 4 (RR = 1.03; 95% CI: 1.00-1.07) and at lag5 (RR=1.05; 95% CI: 1.02-1.08); and among females at lag 2 (RR=1.04; 95% CI: 1.00-1.08). Thus, it was observed that the response among females was different and earlier.

**Table 2.** Correlation matrix for the variables of fine particulate matter (PM_2.5_), temperature (Temp), relative humidity (RH), hospitalizations (Hosp) and male and female sexes. Taubaté, São Paulo, 2011-2012

	PM_2.5_	Temp	RH	Hosp	Male	Female
PM _2.5_	1.00	0.52^*^	0.13^*^	0.04	0.04	0.02
Temp		1.00	-0.07	0.15^*^	0.10	0.13^*^
RH			1.00	0.02	0.08	-0.08
Hosp				1.00	0.86^*^	0.60^*^
Male					1.00	0.12^*^
Female						1.00

^*^P-value < 0.05.

**Table 3.** Coefficients provided by Poisson regression for hospital admissions in relation to fine particulate material concentrations, according to lags (0 to 7 days) and according to sex. Taubaté, São Paulo, 2011-2012

	Both sexes	Male	Female
Lag 0	0.01158 (0.01543)	0.01109 (0.01928)	0.01207 (0.02589)
Lag 1	0.01774 (0.01448)	0.01676 (0.01789)	0.01795 (0.02468)
Lag 2	0.02118 (0.01261)	0.01034 (0.01675)	0.04300 (0.01935)^*^
Lag 3	0.01927 (0.01443)	0.01383 (0.01776)	0.02587 (0.02487)
Lag 4	0.03552 (0.01266)^*^	0.03463 (0.01534)^*^	0.03634 (0.02247)
Lag 5	0.03520 (0.01274)^*^	0.04478 (0.01470)^*^	0.01560 (0.02559)
Lag 6	0.00669 (0.01440)	0.00619 (0.01670)	0.01217 (0.02823)
Lag 7	-0.01189 (0.01335)	-0.00848 (0.01566)	-0.01943 (0.02569)

^*^P-value < 0.05.

In comparing the significant exposures between males and females (at lags 2, 4 and 5) and using equation (1), it was not possible to find any significant differences in the risks shown by the respective coefficients and standard errors. No overdispersion was identified.

**Figure 1.**
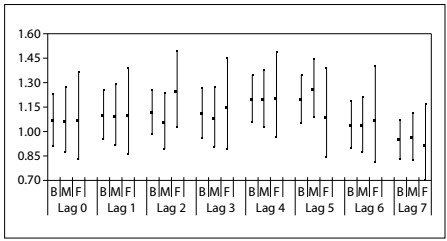
Relative risk with respective 95% confidence intervals,according to a 5 µg/m^3^ increase in fine particulate matter concentration, for both sexes (B) and for males (M) and females (F). Taubaté (SP), 2011-2012.

Figure 1 shows the relative risk values and respective 95% confidence intervals for both sexes and for the male and female sexes separately, according to an increase of 5 µg/m^3^ in PM_2.5_ concentrations. It was evident that the relative risk occurred earlier among females, i.e. at lag 2, without any corresponding occurrence in the analysis on males, or in the analysis on both sexes.

An increase of 5 µg/m^3^ in PM_2.5_ concentration increased the risk of hospitalization for both sexes by up to approximately 18%. The PAR was estimated at approximately 15% and the PAF resulted in an excess of 59 hospitalizations, with expenditure of approximately US$ 150,000.00, assuming an average cost of hospitalization of approximately US$ 2,600.00.

## DISCUSSION

This, to the best of our knowledge, was the first study estimating the effects of exposure to fine particulate matter in a medium-sized city, in relation to the number of hospitalizations due to ischemic heart diseases. The data used were estimated through mathematical modeling and the possible responses to exposure were estimated separately for males and females. An earlier female response was found, occurring two days after exposure (lag 2), but there was no significant difference in relation to the corresponding relative risk among males.

Tuan etal.^14^ examined pollutant concentration data that had been quantified by an environmental agency and found differences in the responses to these pollutants according to the subjects’ sex, regarding the time of occurrence of the hospital admission. In the present study, the data used were estimated through mathematical modeling, whereas Tuan etal.^14^ used data quantified by the environmental agency of the state of São Paulo (CETESB).

Regarding the differences between the sexes, a study on deaths due to ischemic heart diseases that was conducted in all states of the United States between 2004 and 2007 showed that the mortality rate was higher among women than among men, especially among women aged less than 50 years.^15^^,^^16^ We found in our study that there were differences in lag, regarding exposure and hospitalization, and that the effect occurred earlier among females (lag 2) than among males (lags 4 and 5), i.e. four and five days after this exposure, in analyses on both sexes and on the male sex alone after stratification. Among females, this outcome occurred earlier in the study (only two days after exposure), and this result was concordant with the findings of Chen etal.,^17^ who observed that the risk of dying from coronary disease was higher among women, especially among those in the postmenopausal period, than among men.^17^ One possible explanation for the difference in the effects of exposure to PM_10_ and PM_2.5_ between men and women may be that the deposition of these particles is more localized and more intense in females. The smaller number of red blood cells in women may make them more sensitive to the toxic effects of air pollutants.^21^

In studies conducted in Brazil, the effects of exposure to air pollutants have been shown to be associated with hospitalizations due to cardiovascular diseases such as hypertension, acute myocardial infarction and stroke.^6^^,^^18^^,^^19^^,^^20^ Specifically in relation to the association between exposure to particulate matter and hospitalizations due to ischemic heart disease, a study carried out in São José dos Campos, a city near Taubaté, showed that an increase in PM_10_ concentration of 16 µg/m^3^ led to a 10% increase in the relative risk of hospitalization. However, in that study, the subjects were not stratified according to sex.^18^

In the case of cardiovascular diseases, the data estimated through this model in another study made it possible to identify an excess of hospitalizations, of the order of 650, in São José do Rio Preto, that occurred through an increase in PM_2.5_ concentrations, with excess expenditure of US$ 1 million. That study was the only previous study carried out in the state of São Paulo, to the best of our knowledge.^20^

The direct effects of this exposure may occur through agents that cross the pulmonary epithelium in the circulation, such as gases and possibly ultrafine particles (< 0.1 µ), along with soluble constituents of PM_2.5_ (e.g. transition metals). In addition, activation of pulmonary neural reflexes secondary to interactions between particulate matter and pulmonary receptors may play an important role. These direct effects of air pollution provide a plausible explanation for occurrences of rapid cardiovascular responses (within a few hours), such as myocardial infarction. The less acute (several hours to days) and chronic indirect effects of air pollution may occur through pulmonary oxidative stress/inflammation induced by inhaled pollutants. Subsequently, this may contribute towards a systemic inflammatory state, which may, in turn, be able to activate hemostatic pathways, impair vascular function and accelerate atherosclerosis.^25^

The present study had some limitations, and the way in which pollutant concentrations were estimated was one of them. Thiswas done through mathematical modeling and may have provided incorrect data. Another limitation was that the hospitalization data were secondary, even though they came from an official source. Thesedata had the potential to incorporate diagnostic errors or incorrect addresses for the subjects. Additionally, this data source does not contain information on habits such as smoking or sedentary lifestyle, or on comorbidities or family history. It is also important to note that the results presented here do not represent causality, but show an association between the exposure and the outcome. Nonetheless, use of data estimated through the CCATT-BRAMS model, which has already been implemented in other studies, may form an alternative for studies on the effects of exposure to fine particulate matter on human health, such as in relation to respiratory and cardiovascular diseases.^22^^,^^23^^,^^24^^,^^25^

## CONCLUSIONS

Notwithstanding the possible limitations, the findings from this study, using data estimated through a mathematical model, suggest that an association exists between exposure to PM_2.5_ and hospitalizations due to cardiovascular diseases and that this exposure may differ according to sex.
